# Alteration of Interictal Brain Activity in Patients with Temporal Lobe Epilepsy in the Left Dominant Hemisphere: A Resting-State MEG Study

**DOI:** 10.1155/2014/171487

**Published:** 2014-07-21

**Authors:** Haitao Zhu, Jinlong Zhu, Tiezhu Zhao, Yong Wu, Hongyi Liu, Ting Wu, Lu Yang, Yuanjie Zou, Rui Zhang, Gang Zheng

**Affiliations:** ^1^Department of Neurosurgery, Nanjing Brain Hospital Affiliated to Nanjing Medical University, Nanjing, Jiangsu 210029, China; ^2^College of Civil Aviation, Nanjing University of Aeronautics and Astronautics, Nanjing, Jiangsu 210016, China; ^3^MEG Center, Nanjing Brain Hospital Affiliated to Nanjing Medical University, Nanjing, Jiangsu 210029, China; ^4^Department of Medical Imaging, Jinling Hospital, Medical School of Nanjing University, Nanjing, Jiangsu 210002, China

## Abstract

Resting MEG activities were compared between patients with left temporal lobe epilepsy (LTLE) and normal controls. Using SAMg2, the activities of MEG data were reconstructed and normalized. Significantly elevated SAMg2 signals were found in LTLE patients in the left temporal lobe and medial structures. Marked decreases of SAMg2 signals were found in the wide extratemporal lobe regions, such as the bilateral visual cortex. The study also demonstrated a positive correlation between the seizure frequency and brain activities of the abnormal regions after the multiple linear regression analysis. These results suggested that the aberrant brain activities not only were related to the epileptogenic zones, but also existed in other extratemporal regions in patients with LTLE. The activities of the aberrant regions could be further damaged with the increase of the seizure frequency. Our findings indicated that LTLE could be a multifocal disease, including complex epileptic networks and brain dysfunction networks.

## 1. Introduction 

Epilepsy is a common neurological disorder, characterized by hypersynchronous neuronal activity as shown from electrophysiological recordings [1, 2]. Many patients have excellent or good surgical outcomes after the resection of epileptogenic zone. However, the generation and spread of focal onset epileptic seizures involve a large network of brain areas that extended beyond the seizure onset zone (SOZ). Traditionally, the epileptogenic zone was thought to be singular. However, this has been challenged in favor of a network model, in which the focus (or foci) would be widely distributed [3].

During the past decade, there have been an increasing number of studies using structural or functional connectivity methods to research the clinical impact of temporal lobe epilepsy (TLE) on neural networks [4, 5]. Many studies have shown that connectivity abnormalities not only are restricted to the ipsilateral or contralateral temporal lobes, but also involved the extratemporal regions, such as thalamus, cerebellum, frontal lobe areas and cingulate gyrus, and occipital regions [6, 7]. Activity of the regions functionally or anatomically connected to the temporal lobe or hippocampus probably results in complex cognitive and behavioral conditions. These findings have led to the notion of TLE as a “network disease” [3]. Surgical resection is the gold standard for the localization of SOZ and the evaluation of brain function recovery in TLE patients. However, few cases were confirmed by the surgery and histopathological examinations. Furthermore, the relationship between the epileptogenic zone and other altered brain regions in the TLE network is still unclear.

With the development of medical imaging, there are many advanced image techniques used to study the epileptogenic zone and other brain activities. MEG, a noninvasive detection technology, detects neuronal activity directly with millisecond temporal resolution. Compared to electroencephalograph (EEG), which is strongly influenced by conductivity in different organizations within the head, the propagation of magnetic fields is not distorted by the brain, skull, and scalp [8]. Therefore, localizing sources from MEG data is relatively simpler than locating the sources of electric field from EEG data. Previous studies have shown that MEG is a clinically valuable diagnostic tool in presurgical evaluation for both the localization of the epileptogenic zone and the prognosis of surgical outcome [9, 10].

In this study, our hypotheses were as follows: (1) the resting-state brain activity may be different across numerous brain regions, rather than only in SOZs, in left temporal lobe epilepsy (LTLE) patients and healthy controls; (2) these differences could be related to the clinical variables of LTLE; (3) the brain abnormalities of LTLE patients could benefit from surgery of the epileptogenic zone. To confirm SOZs, LTLE patients who planned to undergo surgical treatments were included in our study. Factors such as age at onset (year), seizure frequency (per month), and duration of seizure (month) were recorded and followed up with after surgery. To noninvasively assess resting-state brain activity, resting-state MEG (rsMEG) data were acquired in all subjects and quantified based on the SAMg2 method. The SAMg2 values were compared between two groups to detect altered brain regions in LTLE patients and controls. The correlations were calculated to find the relationships between altered brain regions and clinical records of the LTLE patients.

## 2. Materials and Methods

### 2.1. Subjects

The study was approved by the Medical Ethics Committee of the hospital. Informed consent for the study was obtained from all participants.

From the period of January 2007 to December 2012, 122 patients with refractory epilepsy were admitted to the epilepsy center of the Brain Hospital of Nanjing Medical University (Nanjing, China) and underwent presurgical evaluation. Ninety-eight patients (80.3%) ultimately had cortical resection to treat their epilepsy. Twenty LTLE patients (all right-handed, 7 female and 13 male, mean age 25.25 ± 6.90 yrs) were recruited from the patients who underwent surgical resection for medically intractable epilepsy. Inclusion criteria included (1) seizures with typical temporal lobe semiology that were not controlled with antiepileptic drugs (AEDs); (2) an epileptogenic zone that was located in the left temporal lobe; (3) left hemispheric dominance for language determined by neuropsychological evaluations (etomidate speech and memory test, eSAM, when indicated); (4) patients who underwent surgery for resection of epileptogenic zone; and (5) follow-up time >12 months. General information of the patients is summarized in Table 1.

We assessed the clinical factors including the age at onset (year), seizure frequency (per month), and duration of the seizure (month). The seizure frequency was calculated based on the long-term EEG recordings. Two or more seizures were captured during VEEG monitoring. Seizure semiology, ictal, and interictal EEG data were interpreted by an epileptologist and an electrophysiologist to exclude the pseudoepileptic seizures and other interferences. In our study, the mean monitoring time was 257.70 ± 150.33 hours, ranging from 96 to 764 hours.

Twenty healthy volunteers (all right-handed, 8 females and 12 males, mean age 25.60 ± 5.64 yrs) were recruited as controls, from local community by advertising in the Brain Hospital of Nanjing Medical University. Healthy controls were interviewed and confirmed to have no history of neurological disorders or psychiatric illnesses and no gross abnormalities in brain MRI images. To control the effects of the sleep deprivation in patients, all of our healthy subjects were asked to sleep after 12:00 pm.

### 2.2. Methods

#### 2.2.1. Data Acquisition

All patients had MRI scans with a GE Sigma scanner (GE Healthcare, Milwaukee, WI, USA) before the MEG recording. Three fiducial points were placed in identical locations as the ones used in the MEG recordings so that the 3D MRI and MEG data could be coregistered precisely to yield an MSI using these three landmarks. Three markers, nasion, left ear, and right ear, were stamped on each subject's head. The protocol included the following sequences: axial and sagittal T1 weighted, axial and coronal T2 weighted, axial and coronal fluid-attenuated inversion recovery (FLAIR) images, and three-dimensional (3D) spoiled gradient recalled (SPGR).

rsMEG data acquisitions were performed using a 275-channel whole-head system (CTF VSM MedTech Systems Inc., Coquitlam, BC, Canada) in a magnetically shielded room (MSR) (Vacuumschmelze, Hanau, Germany) that was designed to reduce environmental magnetic noise. Before the MEG scan, there was no reduction in the antiepileptic medication due to the potential risk factor. To increase the likelihood of capturing spike events, we used sleep deprivation. The head position relative to the sensor arrays for each patient was measured using three coils affixed to the nasion and preauricular points before MEG data recording. During MEG recording, all subjects were instructed to rest with their eyes closed and heads still. For each subject, 120-second MEG data was recorded. If the head movement during the recording was greater than 5 millimeters (mm), the epoch was recorded again.

#### 2.2.2. Data Processing

rsMEG data were preprocessed by CTF software (VSM MedTech Systems Inc., Canada, Version CTF-5.2.1). The frequency ranging from 20 Hz to 70 Hz was used in several studies and is outside the range of alpha-band activity that would tend to drive the excess kurtosis negative [11–13]. To eliminate the background activity and contrast the interictal spike activity, we performed SAM(g2) analysis localized the epileptic zone in clinical [12, 14, 15]. First, MEG data were filtered (20–70 Hz). Then, the SAMg2 Z-map of each subject was calculated by the SAMg2 script of the CTF software. After the calculation, the SAMg2 Z-map of each subject was registered with its corresponding 3D MRI by the markers on the nasion, left ear, and right ear. The registered SAMg2 Z-maps were spatially normalized to the Montreal Neurological Institute (MNI) template and resampled to 3∗3∗3 mm^3^. Finally, spatial smoothing was conducted on the Z-maps with an isotropic Gaussian kernel of 6mm of full width at half maximum.

#### 2.2.3. Statistical Analysis

One way analysis of covariance (ANCOVA) with age and gender as covariates was used to compare SAMg2 maps of healthy controls and those of LTLE patients. The voxel-wise ANCOVA *P* values were less than 0.05 after false discovery rate (FDR) correction was considered significant.

According to the voxel-wise significant differences between two groups, regions with suprathreshold clusters were defined as regions of interests (ROIs) for further analyses. Mean SAMg2 *Z* values of these regions were calculated using Automated Anatomical Labeling atlas [16]. Correlations between ROIs and clinical variables of LTLE (seizure onset, seizure frequency, and duration of seizure) were calculated. All data were shown as mean ± SD. An FDR-corrected *P* values less than 0.05 were considered significant.

## 3. Results

There was no significant difference in age and gender between the two groups (*P* > 0.05).

### 3.1. Brain Regions with Abnormal Brain Activity between Two Groups

**Figure 1:**
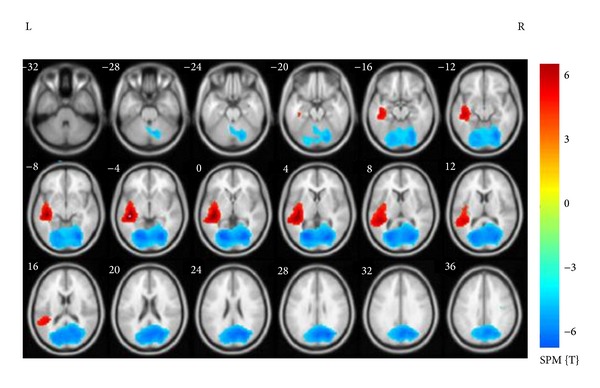
Statistical parametric maps depict SAMg2 increases (warm colors) and decreases (cool colors) in LTLE compared with controls. Significant increases occur in left side hippocampal gyrus, insula, superior temporal gyrus, middle temporal gyrus, inferior temporal gyrus, and Heschl gyrus, while decreases occur in the bilateral calcarine, cerebellum, cuneus, fusiform, lingual gyrus, occipital lobe, precuneus, left parietal gyrus, and contralateral precentral gyrus.

In the LTLE patients compared to gender and age matched healthy subjects, there was a significant reduction in SAM g2 signals in the bilateral calcarine, cerebellum, cuneus, fusiform, lingual gyrus, occipital lobe (superior, middle, and inferior gyrus), precuneus, left parietal gyrus, and contralateral precentral gyrus. Patients also showed increased SAM g2 signals in the ipsilateral (left side) hippocampal gyrus, insula, superior temporal gyrus, middle temporal gyrus, inferior temporal gyrus, and Heschl gyri (*P* < 0.05, FDR corrected) (Table 2 and Figure 1).

### 3.2. Correlation Analysis of Abnormal Activity and Clinical Variables

The relationship between the above abnormal regions and clinical variables (age at seizure onset, frequency of seizures, and duration of epilepsy) was evaluated using multiple linear regression analysis. We found that multiple abnormal regions were significantly associated with frequency of seizures (Table 3). Duration of epilepsy and age at seizure onset did not significantly influence brain activity.

## 4. Discussion

In this study, the difference in the SAMg2 signals between patients with LTLE and healthy subjects was used to measure the disruption of neuronal activity in epileptic and distant brain regions during the interictal period. Significant differences were observed in the spatial pattern and intensity of SAMg2 signals in the two groups. These findings are discussed further below. The special changes of the resting-state brain activity may be potential noninvasive biomarkers for understanding, diagnosing, and potentially treating the LTLE patients.

Compared to the healthy control group, SAMg2 values increased in the ipsilateral (left side) hippocampal gyrus, insula, superior temporal gyrus, middle temporal gyrus, inferior temporal gyrus, and Heschl gyri of the patients. These results revealed that the irritated areas were beyond the SOZ but restricted to the ipsilateral temporal lobe and mesial structure. We propose that the increased SAMg2 signals in specific regions may form irritated areas that might be responsible for seizure genesis and propagation. The removal of the irritated areas may be an effective treatment for the LTLE patients.

The role of the neurosurgeon in the surgical treatment of TLE had evolved from doing so-called standard anterior temporal lobectomies (TLR) to selective amygdalohippocampectomy (SAH) and temporal resection guided by intraoperative electrocorticography. A number of studies were devoted to the comparison of seizure results from temporal lobectomy (TLR) with selective amygdalohippocampectomy (SAH) [17, 18]. And several studies have reported that seizure outcomes are irrespective of the extent of mesial and lateral resection [19, 20], while others state that seizure outcomes are better if larger volumes or specific substructures are removed [21]. Comparing TLR and SAH, McKhann pointed out, “neither SAH nor TLR could be recommended over the other option as a standard or guideline in the surgical management of TLE” [22]. The Spencer type of resection [23], which consists of a small anterior partial lobectomy combined with a more extensive mesial resection, may be an ideal method for the treatment of TLE. Our study reveals that the irritated areas of LTLE involve not only the lateral neocortical structures but also the ipsilateral nearby structures, such as the hippocampus (HC), insula, and Heschl gyri. These interictal irritated zones may be involved in the LTLE network formation. The Spencer type of resection would be sufficient enough to resect or interrupt a large enough part of the mesial structures to render the mesiobasal network between the HC, parahippocampal gyrus, and amygdalum unable to build up and sustain a seizure. Abosch et al. [24] referred to this as “severing a critical proportion of the connections” when they discussed the variability in extent of resection described by McKhann et al. [22].

In this study, all of the patients were diagnosed with LTLE and eleven of them underwent the Spencer type of resection (left anterior temporal lobectomy combined with partial amygdalohippocampectomy). The seizure freedom (Engel class I) of them was 72.7% (8/11) better than the group of the remainder 55.6% (5/9). The results from this study have further proved that the refractory LTLE is not a focal disease but a network disease and can be cured by extensive surgical resection.

The appearance of the insula is also significant because of its particular interconnection with the amygdalohippocampal structures. Isnard et al. found that seizure activity invaded the insular cortex in all temporal lobe epilepsy patients studied with chronic depth stereotactic recordings [25]. This may explain the persistence of seizures after selective amygdalohippocampectomy. This study also had its own limitations; we have removed the anterior temporal lobe and the mesial structures except the insula during surgery.

The reduction in SAMg2 values is thought to reflect deactivated brain activity or dysfunction in the special regions. Compared with healthy controls, LTLE patients showed multifocal dysfunction in the bilateral calcarine, cerebellum, cuneus, fusiform, lingual gyrus, occipital lobe (superior, middle, and inferior gyrus), and precuneus brain regions. The ipsilateral superior parietal gyrus and contralateral precentral gyrus also displayed reduction. The deactivated areas extend widely beyond the ipsilateral hemisphere to the contralateral or bilateral areas in patients with LTLE.

A relatively large dysfunctional area was observed in the bilateral visual cortex, including the bilateral calcarine, fusiform, lingual gyrus, and occipital lobe (superior, middle, and inferior gyrus) in this study. Van Paesschen et al. used SPECT to study the cerebral perfusion changes in complex partial seizures patients with hippocampal sclerosis. They found bilateral occipital hyperperfusion during complex partial seizure and relative occipital hypoactivation during interictal period [26].

The cause of interictal bilateral visual cortex dysfunction is unknown, but propagating epileptiform discharges through the branches of the inferior longitudinal fasciculus (ILF) may play a role. Catani et al. [27] used tractography in the living human brain to address the connections between occipital and temporal lobe and found direct fiber connections between the occipital and anterior temporal cortex in a bundle labeled the ILF. In our study, the dysfunctional regions also involved the three origin branches of the ILF: a lingual branch, a lateral occipital branch, and a cuneal branch. Moreover, a recent study combining fMRI and tractography has visualized the propagation of epileptic activity from the temporal epileptogenic focus to the occipital lobe in mesial temporal lobe epilepsy [28]. The involved cortices lie along the occipitotemporal connections supplied by the inferior longitudinal fasciculus, suggesting a direct propagation pathway from the anterotemporal to the occipital lobe [29]. However, in line with previous brain imaging studies we take our data to suggest that the left side epileptic interictal discharges may influence the activity of the bilateral visual cortex in the patients with left temporal lobe epilepsy.

Our study just focuses on the reduced active regions of the default mode network (DMN) within the bilateral precuneus, ipsilateral superior parietal gyrus, while the other regions of the DMN are normal. Decreased SAMg2 signals within the DMN are considered to result from the disruption of neuronal activity and are commonly used to reflect associated impairments in brain disorders. The lateral parietal cortex is involved in spatial attention aspects of word reading [30]. The precuneus (PCUN) has been reported to be involved in consciousness, engaged in self-related mental representations during rest [31], and related to the late recovery of consciousness in epilepsy patients [32]. The posterior cingulate cortex/precuneus (PCC/ PCUN) is activated during tasks that involve autobiographic memory and self-referential processes [33]. TLE patients often present a few abnormal psychological and psychiatric symptoms associated with the functionalities of the DMN [34], such as absence of self-awareness, emotional and psychic experiences, and social cognitive impairments.

The motor impairment of LTLE patients has been demonstrated by this study. Our MEG study revealed that the deactivated region involved the contralateral precentral gyrus and the bilateral cerebellum. The contralateral precentral gyrus is dominated by the control of limb movement ipsilateral to the epileptic focus, while the cerebellum is by sensory-motor integration, motor coordination, and so forth. Nelissen et al., using SPECT and PET to study the brain blood perfusion in TLE, found interictal hypometabolism in the frontal lobe cortex. They suggested a dynamic process of frontal lobe function inhibition, which not only could represent protection against epileptiform-discharge propagation, but could also be responsible for the functional deficits presented by these patients. In this study, we also found more serious damages in bilateral cerebellum lobe in our LTLE patients. This phenomenon was so-called “crossed cerebellar diaschisis” and was considered an indication of disconnection of the glutamatergic corticopontocerebellar tracts [35]. The decreased SAMg2 values of the bilateral cerebellum lobe could be a sign of decreased motor coordination.

In our study, sleep deprivation was used to increase the likelihood of capturing spike events which were performed in all epilepsy patients in our epilepsy center. Many studies also adopted the similar sleep deprivation method to increase the likelihood of capturing spike events in clinical [36, 37]. A lack of sleep has been demonstrated to produce performance deficits in experimental tasks of alertness, attention, memory, cognition, learning, and motor responses [38]. While the neurophysiological effects of sleep deprivation remain incompletely understood, a few recent studies have begun to provide guidance on where and what neurophysiological changes occur as a function of sleep deprivation. Thomas et al. quantified and characterized global and regional brain activity changes implicated in sleep deprivation-induced neurobehavioral impairment during cumulative, extended sleep loss [39]. Significant decreases in CMRglu were reported in the thalamus, prefrontal and posterior parietal cortices. Alertness and cognitive performance declined in association with these brain deactivations. This study provides evidence that short-term sleep deprivation produces global and regional decreases in brain activity, with larger reductions in activity in the distributed cortical-thalamic network mediating attention and higher-order cognitive processes. The results from sleep deprivation were different than the hyperactivity and dysfunctional regions which were due to epilepsy in our study. In our study, the healthy volunteers were demanded to sleep after twelve o'clock before MEG examinations to control the additional effects of the sleep loss in LTLE patients. The decreased brain activities detected in our patients were caused by the long-term effects of epilepsy rather than sleep loss. Considering the complex effects of sleep deprivation on brain activity, further study should be performed to control sleep loss in patients with epilepsy.

In our study, we used the SAMg2 signals to study how brain activity in LTLE patients differs from brain activity in healthy controls. Specifically, seizure frequency was strongly associated with intensity of abnormal activity in the LTLE patients. In the study, we further confirmed that TLE is not a focal focus, but a multifocal disease. The mechanism of these extensive abnormal activities needs further study.

## 5. Limitations 

In the study, we did not compare the preoperative brain activities of TLE with the changes of postoperative patients. Further, this study was limited by not comparing abnormal activities with neuropsychological findings and prognostic factor. To make up the difference between TLE patients and controls in the sleep loss, all our health subjects were asked to sleep later. Though sleep loss was not totally controlled, our findings were quite different from those in sleep deprivation, indicating that our study was less affected by sleep deprivation. However, it is difficult to exclude the potential impact of the sleep deprivation to the brain activity of the patients and the normal subjects. Further study should be conducted to specify the effects of sleep loss in TLE patients.

## Figures and Tables

**Table 1:** Demographic and clinical characteristics of the patients.

Number	Age (Y)/gender	Age at onset (Y)	Seizure frequency/Mo	Duration of seizure (Mo)	MRI (lesion location)	Interictal MEG (SAMg2)	EEG (intro/extroperation)	Surgical procedure	Pathology	Follow-up (months)/outcome (Engle)
1	35/M	23	4	12	LT	LT	LT	Lesionectomy + ATL	LT cortical dysplasia	35/IA
2	30/M	14	25	16	NM	LT	LT (ant)	ATL	LT neurodegeneration	46/IA
3	23/F	13	5	10	LT	LT, LH	LT (ant, bas-lat)	ATL + SAH	LT neurodegeneration; LH HS	35/IA
4	21/M	4	3	17.5	LT, LH	LT, LH	LT (bas-lat)	ATL + SAH	LT gliosis; LH Atrophy	29/IIA
5	36/M	32	4	4.5	LT	LT	LT (lesion-pos)	Lesionectomy + ATL	LT angiomatosis	39/IA
6	21/F	6	5	15	LT	LT	LT (ant, bas-lat)	ATL + SAH	LT neurodegeneration; LH atrophy	65/IB
7	32/F	22	12	10.5	NM	LT, LH	LT (ant, bas-lat)	ATL + SAH	LT neurodegeneration; LH atrophy	24/IA
8	18/M	9	5	9	LT	LT	LT (lat, mid-inf)	Lesionectomy + ATL	LT ganglioneuroma	60/1B
9	38/F	30	30	8.5	LT	LT	LT (perilesion)	Lesionectomy + ATL	LT cortical dysplasia	58/1A
10	22/F	2	4	20	NM	LFT	LAT	ALT	NM	37/IIA
11	25/M	16	7	9.5	LT	LT	LT (lesion-pos)	Lesionectomy + ATL	LT cortical dysplasia	26/IB
12	29/M	8	11	21	NM	LT	LAT	ALT	LT neurodegeneration	17/IIIB
13	16/M	2	3	14.5	LT, LH	LT, LH	LT (ant, bas-lat)	ATL + SAH	LT neurodegeneration; LH atrophy	22/IA
14	22/F	6	15	16	LT	LT	LT	Lesionectomy + ATL	LT cortical dysplasia	44/IA
15	21/M	11	22	10	LT, LH	LT, LH	LT (ant, bas-lat)	ATL + SAH	LT gliosis; LH atrophy	16/IA
16	33/M	27	28	6	LT, LH	LTP, LH	LT (ant, bas-lat)	ATL + SAH	LT neurodegeneration; LH HS	21/IA
17	16/M	4	6	12	NM	LT, LH	LT (ant, bas-lat)	ATL + SAH	LT gliosis; LH atrophy	15/IA
18	25/M	13	8	12	LT	LT	LT	Lesionectomy + ATL	LT cortical dysplasia	33/IA
19	16/F	3	2	13	LT, LI	LT, LI	LAT, LI	ATL + SAH	LT neurodegeneration; LI atrophy	38/IIC
20	26/M	15	6	11	NM	LFT	LT (perilesion)	Lesionectomy + ATL	LT cortical dysplasia	49/IB

M: male; F: female; LT: left temporal; NM: normal; LH: left hippocampus; LI: left insular; LFT: left frontal-temporal; ant: anterior; bas: basal; lat: lateral; mid: middle; pos: post; inf: inferior; ATL: anterior temporal lobectomy; SAH: selective amygdalohippocampectomy; HS: hippocampal sclerosis.

**Table 2:** Brain regions with increased and decreased activations in the patient group.

Brain ROI	Voxels	*T* value	MNI coordinate		
			*X*	*Y*	*Z*
Temporal_Mid_L	270	4.15	−48	−33	0
Hippocampus_L	75	4.11	−36	−27	−6
Temporal_Sup_L	179	4.1	−45	−36	6
Temporal_Inf_L	36	4.04	−42	−30	−12
Heschl_L	38	3.96	−39	−21	6
Insula_L	87	3.89	−39	−18	0
Fusiform_L	92	−2.03	−24	−75	−3
Precentral_R	68	−3.67	45	−3	51
Cerebelum_L	310	−3.71	−18	−72	−12
Cerebelum_R	424	−3.82	24	−81	−18
Parietal_Sup_L	38	−3.86	−15	−69	42
Precuneus_L	242	−4	0	−66	30
Occipital_Sup_L	210	−4.03	−18	−81	12
Occipital_Inf_R	129	−4.04	30	−81	−6
Occipital_Inf_L	115	−4.06	−27	−81	0
Occipital_Mid_L	295	−4.07	−27	−75	0
Precuneus_R	405	−4.14	15	−66	24
Occipital_Mid_R	262	−4.15	27	−72	27
Lingual_R	554	−4.19	24	−75	−3
Calcarine_L	533	−4.3	−21	−72	6
Occipital_Sup_R	226	−4.32	27	−69	27
Cuneus_L	322	−4.33	0	−72	24
Lingual_L	456	−4.37	−21	−72	3
Fusiform_R	159	−4.44	27	−75	−6
Calcarine_R	474	−4.44	24	−75	9
Cuneus_R	335	−4.49	21	−69	27

L: left; R: right; Mid: middle; Sup: superior; Inf: inferior.

**Table 3:** Results of correlation analysis between the abnormal activity and the seizure frequency (*P* < 0.05 with FDR correction).

Brain ROI	*P* value	*R* value
Precentral_R	0.025	0.377
Hippocampus_L	0.025	0.406
Calcarine_L	0.025	0.373
Calcarine_R	0.027	0.349
Cuneus_L	0.021	0.474
Cuneus_R	0.026	0.357
Lingual_R	0.026	0.409
Occipital_Sup_L	0.031	0.488
Occipital_Sup_R	0.021	0.454
Occipital_Mid_L	0.025	0.383
Occipital_Mid_R	0.040	0.520
Occipital_Inf_L	0.022	0.441
Occipital_Inf_R	0.023	0.478
Fusiform_L	0.027	0.481
Fusiform_R	0.026	0.388
Parietal_Sup_L	0.022	0.431
Precuneus_L	0.022	0.459
Precuneus_R	0.027	0.389
Temporal_Mid_L	0.024	0.370
Temporal_Inf_L	0.033	0.577
Cerebelum_L	0.035	0.313
Cerebelum_R	0.028	0.517

L: left; R: right; Mid: middle; Sup: superior; Inf: inferior.
